# Adolescent Pregnancies and Perinatal Mental Health—Needs and Complex Support Options: A Literature Review

**DOI:** 10.3390/jcm14072334

**Published:** 2025-03-28

**Authors:** Sigita Lesinskienė, Justina Andruškevič, Agnė Butvilaitė

**Affiliations:** Clinic of Psychiatry, Institute of Clinical Medicine, Faculty of Medicine, Vilnius University, 01513 Vilnius, Lithuania

**Keywords:** adolescent pregnancy, mental health, complex needs, support options

## Abstract

Adolescent pregnancy remains a global issue, demanding comprehensive, long-term solutions. Despite declining rates, early pregnancy leads to severe physical and mental health risks along with increased mortality. Therefore, adolescent pregnancy requires urgent global action. This literature review evaluates pregnant adolescents’ psychological health issues, explores their needs, and investigates interdisciplinary approaches to enhance mental and physical health support. Studies show that adolescent pregnancy poses significant mental health risks and is associated with high rates of depression, suicidal ideation, low self-esteem, substance use, and anxiety. Co-occurring hardships further worsen psychological well-being. Found data indicated that the main needs of pregnant adolescents were adequate sexual and reproductive health; legal rights; high-quality, accessible healthcare; and socioeconomic support. The included references revealed several interventions and recommendations for supporting pregnant adolescents while highlighting challenges in the current framework. Pregnant adolescents face diverse gaps in current support systems. Further research is needed addressing social services, reproductive health consultations, and mental health support. Greater emphasis on multidisciplinary approaches and examples of effective support strategies is crucial to creating a nurturing environment and securing the well-being of pregnant adolescents.

## 1. Introduction

Pregnancy among girls under the age of 19 remains a serious problem; despite decreasing rates, it still demands complex, long-term solutions [1]. At the outset, pregnancy during early adolescence brings tremendous implications for girls’ physical health, as their bodies may not be prepared for such physiological changes. Young mothers are at higher risk of poor nutrition, anemia, premature membrane rupture, and eclampsia. Other potential risks include obstetric fistula, puerperal endometritis, miscarriage, systemic infection, and a high possibility of bleeding. The highest risk of severe perinatal complications is considered to occur when pregnant during the first 2 years after menarche. Pregnancy-related conditions rank among the leading five causes of disability-adjusted life years and mortality in the adolescent girl population [2,3,4]. The mortality rate among adolescent mothers is concerned to be more than one-third higher compared to adult women [5]. Although still high, the adolescent pregnancy rate has been steadily decreasing over the past 15 years, driven by improved access to education, contraception, and other prevention strategies [4]. However, despite this progress, the adolescent fertility rate in Africa remains more than four times higher than in high-income countries [5]. Teenage mothers-to-be have a higher likelihood of detecting pregnancy at a later stage. In addition, girls encounter challenges in identifying whether the unexpected pain is caused by acute abdomen or labor, ultimately exposing themselves to danger and delivering at home. Moreover, adolescents tend to use alcohol, tobacco, or other psychoactive drugs while expecting, which affects both mother and child [4,6]. Evidence suggests that most adolescent pregnancies are unanticipated and approximately 50% of them lead to abortion [4]. However, if the decision to give birth has been made, the well-being of adolescent mothers’ babies is under threat as well. There is a higher risk of nutritional problems, stunting, preterm delivery, asphyxia, low birth weight, or stillbirth [3]. Infants born to adolescent mothers have a 50% higher mortality rate [5]. Moreover, newborns of teenage mothers have a higher probability of having lower Apgar scores and being admitted to an intensive care unit [7].

All problems experienced by underage pregnant women can lead to a deterioration of psychological health [8]. Younger maternal age is associated with a higher risk of postpartum depressive disorder and higher levels of stress and anxiety. Generally, the prevalence of postpartum depression in developed regions is approximately 13–19%, whereas in the adolescent population, it reaches 40% [9]. Pregnant adolescents before the age of 16 are particularly vulnerable and are more likely to experience mental health issues than their non-pregnant peers [10]. Moreover, adolescent pregnancy brings up individual and social challenges concerning human rights protection along with socioeconomic impacts [3]. Adolescent mothers face the huge stress of being financially deprived and having limited education or employment opportunities. Some pregnant adolescents are stigmatized, forced to run away from their houses, experience abuse, and face a lack of support from family members or the child’s father [11].

Together with child marriage prevention, adolescent pregnancy reduction is a part of the Sustainable Development Goals agenda established by the United Nations [12]. Global, regional, and national measures to support vulnerable groups are constantly expanding [12]. By implementing large-scale programs, more and more countries are encouraged to take urgent action [12]. It is important to ensure further investigation and enhance adolescent-friendly services supporting mental and physical health during and after pregnancy [13]. This review aims to assess mental health issues, identify complex needs, explore ways to organize comprehensive perinatal assistance and determine deficiencies in the existing support system for teenage pregnant women. This will provide foundations for organizing interdisciplinary mental health support in regions where pregnant minors do not receive adequate assistance. Additionally, it will contribute to a profound understanding of the mental health needs of young mothers for psychologists, social workers, physicians, and healthcare policymakers.

## 2. Methods

A literature review was conducted using the electronic scientific database PubMed, covering the period from 2015 to 2025. The terms applied in PubMed Advanced Search included “Adolescent pregnancy” OR “Pregnancy in Adolescence” OR “Teen pregnancy” OR “Teenage pregnancy” AND “Complex support” OR “Healthcare” OR “Patient care” OR “Psychosocial support” OR ”Social support” OR “Community health services” OR “Primary care interventions” AND “Mental health” OR “Psychological well-being”. Additional literature was included after cross-referencing publications retrieved through a Boolean search. Demographic trends were estimated using supplementary data from the United Nations, World Health Organization (WHO), UNICEF, National Center for Health Statistics, and Lithuanian Institute of Hygiene. Several studies were manually identified in the PubMed and Google Scholar databases using the keywords “Prevalence”, “Rate”, and “Adolescent pregnancy” in Lithuanian and English. Our eligibility criteria were full-text articles available for analysis, written in English or Lithuanian, and published between 2015 and 2025. The following exclusion criteria were applied: written in languages other than English or Lithuanian, the referenced cohort or subgroup not classified as adolescents, and the study having been conducted with species other than humans. All authors participated in this research, engaging in a thorough discussion of the selected articles, critically reviewing the research methodology, assessing the topic’s relevance, and systematically analyzing abstracts before proceeding with a comprehensive evaluation of the full texts.

## 3. Search Results

**Figure 1:**
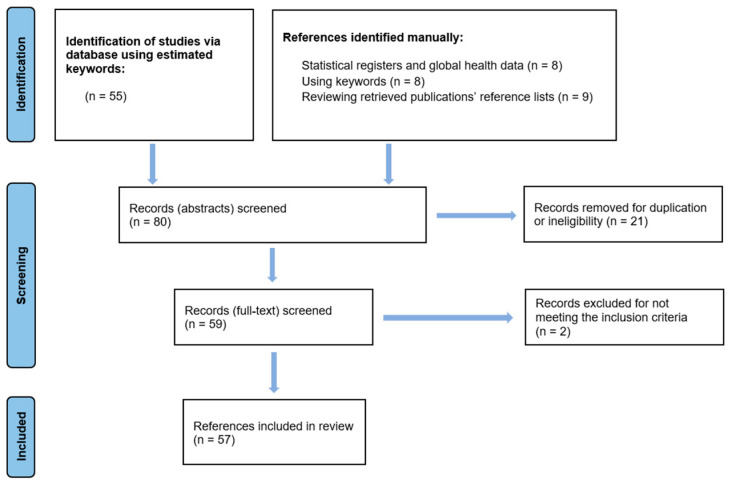
PRISMA flow diagram.

Using the estimated Boolean query, 55 articles were found and reviewed for potential inclusion. An additional 25 records were identified through manual searching and cross-referencing. In total, 80 records were screened. Twenty-one studies were eliminated due to ineligible abstract content or duplication. Two more articles were excluded because of the unavailability of the full text. A total of 57 references fulfilled the eligibility criteria and were included in this review (Figure 1).

### 3.1. The Prevalence of Adolescent Pregnancy

Before addressing pregnant adolescents’ mental health issues, needs, and support options, it is essential to understand the scale of the problem. We carefully analyzed found data and included prevalence rates from diverse regions around the world. As authors from Lithuania, it was essential for us to analyze the matter of adolescent pregnancy in our country and incorporate data from Lithuania alongside global statistics. Adolescent pregnancy is a widespread issue. According to the WHO statistical data, the birth rate among 15-to-19-year-old adolescents was 51.2 per 1000 girls in 2013, decreasing to 41.3 births per 1000 in 2023. The birth rate among 10-to-14-year-olds was 2 in 2013 and decreased to 1.5 in 2023 [14]. On a global scale, approximately 13% of adolescents gave birth before reaching adulthood in 2023, indicating that almost one in six teenage girls gave birth before the age of 18 [2]. In 2016, 2.03% of American women aged 19 and younger gave birth. Among all births in the United States that year, 5.3% were to teenage mothers. The highest birth rates among women aged 15 to 19 were observed in American Indian/Alaska Native (3.51%) and Hispanic women (3.19%), while Asian American (0.39%) and White women (1.43%) had the lowest rates [15]. The adolescent birth rate varies by state in the United States of America. According to the statistical data, in 2022, it ranged from 4.6 births per 1000 girls in New Hampshire to 26.4 births per 1000 in Mississippi [16]. A scoping review conducted in 2023 found that the rates of adolescent pregnancy among African, Caribbean, and Black North Americans were almost three times higher than among White teens [17]. In Turkey, the adolescent birth rate ranges from 2% in the western regions to 7% in the southern parts of the country [18]. A study of socioeconomically underdeveloped regions of Turkey found that out of 16,985 pregnant women, 1719 were adolescents, which resulted in a prevalence rate of ~10% [19]. By analyzing the Medical data of Births in Lithuania, a decrease in adolescent pregnancies can be observed: 93 girls gave birth before the age of 18 in 2022, whilst in 2012, the number of pregnant adolescents reached 356 [20,21]. Referring to research conducted by the Lithuanian team, the adolescent birth rate in Europe was 10.8 per 1000 in 2015, with the lowest rates in the Netherlands (3.2), Denmark (3.4), Sweden (4.5), Cyprus (4.9), and highest rates in Romania (36.6) and Hungary (41.3) [22]. Adolescent pregnancy prevalence in the Eastern Mediterranean region reaches 9% [23]. In Bangladesh, Angola, Mozambique, and Nigeria, there are approximately 10 pregnancies per 1000 girls aged only 10–14 [24]. Nearly one in five pregnancies in rural South Africa occurs before the age of 20 [10]. Analyzing the data of Africa, the pooled percentage of pregnant adolescents was 18.8% with the highest prevalence observed in the East African sub-region (21.5%) and the lowest in Northern Africa (9.2%) [25]. While the global adolescent birth rate has declined, it remains an issue since the pace of change has been inconsistent across different regions. Additionally, there are substantial variations in the rates between and within countries [12]. The problem of teenage pregnancy is still highly severe in low- and middle-income areas. The improvement remains slow in Sub-Saharan Africa, East Asia, Latin America, and the Pacific regions [2,3,12,22]. Relevant studies on the prevalence of adolescent pregnancy are presented in Table 1.

### 3.2. Adolescent Perinatal Mental Health Issues

Unplanned pregnancy in individuals aged 10–19 is a significant factor in the deterioration of psychological well-being. Pregnant adolescents are cognitively and emotionally immature and experience perinatal mental health issues at rates up to 30% higher compared to their non-pregnant peers and adults [26,27,28]. Mental health struggles can arise from numerous risks, such as experiencing different forms of abuse or being exposed to community stigma. Other factors include having HIV, lacking necessities, and being in a relationship with a much older or younger partner who is the father of their child. Facing abandonment, unfulfilled ambitions, prior psychological health issues or traumatic events, low educational level, and economic hardships further contribute to the higher level of psycho-emotional distress and deteriorate adolescent mental health [9,26,29,30,31,32]. When discussing mental health challenges faced by pregnant adolescents, it is important to note that those with mental health disorders are more likely to engage in risky sexual behavior compared to their peers without such disorders [29]. As a result, this can contribute to unplanned pregnancies among teenage girls. In another separate study, researchers found that two-thirds of adolescents with mental disorders had been sexually active, one-quarter had experienced a previous pregnancy, and one-fifth tested positive for STIs [33]. The key perinatal mental health issues identified in articles found in databases are discussed below (Table 2).

#### 3.2.1. Depression

Depression is a mental health condition that many mothers under 19 struggle with. Although it is uncommon in childhood, its prevalence rises sharply during adolescence [34]. The components identified as being associated with perinatal depression in pregnant adolescents were young age, social isolation, low self-esteem, unemployment, and food deprivation [26,28]. An additional determinant of depressive disorder in young mothers is early marriage and life in the extended family [35].

Rahim K. A. and his team conducted a systematic review and meta-analysis, revealing that depression together with anxiety were the most widespread mental health issues among adolescent mothers postpartum [36]. In Kenya, research showed that one in four adolescent mothers experienced postpartum depressive symptoms. The study’s multivariable analyses suggested that both parental encouragement for girls’ education and having a close female friend to talk to were protective factors against depression [11]. A cross-sectional study of pregnant Jordanian adolescents showed that almost 29% of young mothers have a high probability of postpartum depression [35]. Another study conducted in Mexico estimated that a little more than half of adolescent mothers experienced depression during pregnancy [37]. The rate of youths’ perinatal depression between 30 primary maternal and child healthcare centers in Ibadan, Nigeria, was 18% [38]. Another study indicated that around 37% of Ethiopian adolescents suffer from postpartum depression, while the percentage rate among adults was 20% [26]. A high prevalence of mental health issues among adolescents during the peripartum period was observed in other studies [31,32].

Unintended pregnancy and the resulting postpartum depression can negatively affect an adolescent mother’s capacity to care for and engage with her baby. This can lead to premature infant death and violation of children’s fundamental rights. The condition contributes to a negative perception of motherhood, parental neglect, and an insecure attachment between mother and child [9,26]. However, only a small percentage of pregnant girls and young mothers with depression seek professional help (25%) or use mental health treatment (15%) [39].

#### 3.2.2. Suicide

Adolescent mothers are more prone to experiencing suicidal thoughts and making suicide attempts. Regardless of whether they were pregnant, suicidal ideation and attempts continue to be a major issue among adolescents [32,36]. Global systematic reviews have reported a broad variation in suicidal ideation, ranging from 3% to 33% worldwide, with mothers aged 20 or younger facing significantly higher odds of attempting suicide compared to adult women [40].

#### 3.2.3. Low Self-Esteem

Becoming a mother before the age of 19 can lead to feelings of isolation, hopelessness, and emotional turmoil [11]. Adolescent mothers have more negative attitudes towards the physical changes they experience during pregnancy and face the unfulfilled expectations of motherhood. Young expectant mothers experience self-criticism and feelings such as worthlessness and shame. Balancing the desire for independence as a growing adolescent with the need for support as a young mother can cause internal conflict and further contribute to the increased risk of poor mental health [6,28].

#### 3.2.4. Using Substances

There is evidence that adolescents are involved in high-risk behaviors. In a study focused on pregnant Mexican American adolescents, when asked about substance use, most teens viewed it as a way to temporarily relieve stress. While many disapproved of substance use, recognizing its harm to both mother and baby, substances like marijuana and alcohol were still used to cope with perinatal depression [39]. This is problematic because they can worsen depressive symptoms during the perinatal period. Additionally, psychotropic substance abuse and conduct problems double the probability pregnancy in adolescence [17].

#### 3.2.5. Anxiety

In a study examining the link between perceived social support and anxiety in pregnant adolescents, it was found that 13.6% of the participants experienced some form of anxiety disorder. The most common disorder was generalized anxiety disorder (GAD), affecting 8.7% of the group; followed by social phobia (4.8%); obsessive–compulsive disorder (OCD) (3.7%); post-traumatic stress disorder (2.4%); and panic disorder (2.1%). Women who had social phobia, OCD, or GAD reported lower levels of social support across types of social support areas [41]. Participants in the study conducted by Judith Osok and her team shared how adolescents during the perinatal period were overwhelmed by feelings of shame, anxiety, and fear. Some even contemplated denying their pregnancies, despite obvious signs such as missed periods for several months. Moreover, adjusting to a new relationship with their mothers-in-law added to the stress within the family, even though in-laws were described as both sources of stress and support [42,43]. Additionally, during pregnancy, girls expressed concerns about relationships and fears of being unprepared for labor and parenting [43].

### 3.3. Complex Needs of Adolescent Mothers

Analysis of the data from the included references highlighted the common needs of pregnant adolescents. In Table 3, we provide a concise summary of categorized complex needs, supplemented by the number of references found.

#### 3.3.1. The Need for Adequate Sexual and Reproductive Health and Rights (SRHR)

Adolescents have unique requirements concerning sexual and reproductive rights and health. Disregarding these specific needs can result in detrimental outcomes [44]. Adolescent pregnancy is often driven by a range of factors, including early sexual activity, rapid physical development, risky sexual behaviors, having multiple partners, inadequate reproductive health education, and misuse or lack of contraception [45]. The use of contraception is especially significant in groups at high risk for pregnancy, such as adolescent mothers, who are at an increased risk of subsequent pregnancies—about 40% experience another pregnancy within two years [46].

Moreover, adolescent pregnancy is associated with gender-based violence and emotional or sexual abuse [28,42,45]. This is compounded by the fact that many pregnant adolescents report that perpetrators of sexual violence are often schoolmate boyfriends or peers. This highlights the alarming reality that sexual violence can occur within relationships that young girls trust, making the need for contraceptive access and education even more crucial. The fact that pregnant or parenting survivors are often hesitant to disclose sexual violence reveals a gap in understanding or awareness around consent and healthy relationships [47]. As an example, approximately 40% of girls before the age of 18 experienced non-consensual sexual intercourse in Zimbabwe [44].

There is a critical need for education that promotes healthy sexual relationships, helps young girls to recognize their rights and understand what constitutes abusive behavior, and equips them with the tools to speak up if they face violence. However, some cultures are deeply religious and uphold conservative views on nonmarital pregnancy, sexual education, and contraception, which restricts open discussion about reproductive health services and makes it harder to implement changes [48].

#### 3.3.2. The Need for High-Quality and Accessible Healthcare Possibilities

For White women in America, access to birth control and abortion services was a key reproductive rights issue during the rise of the feminist movement. Today, young pregnant women still face similar challenges and needs [49]. Unintended pregnancy puts young girls at higher risk of health adversities. Therefore, it is crucial to ensure high-quality preconception preventive care. Although adolescents are twice as likely to receive guidance in mental health issues and four times more likely to be educated about vaccination in primary care, they still lack comprehensive counselling on unexpected pregnancy [50].

Acknowledging their high vulnerability to psychological health issues, pregnant adolescents critically require advanced mental health services and comprehensible tools [27,44,51]. According to a study, the second most common need of peripartum adolescents in Sub-Saharan Africa was addressing the mental health burden, with depression being the most prevalent of all psychiatric disorders (71.4%) [52]. Following the data collected in 2017 from the Mental Health Atlas project, almost half of African states have no designated mental health funding, and 20% of them lack formal psychological well-being legislative provisions [29].

Yet there are still regions in the world where proper care standards and accessibility appear diminished among adolescent girls compared to women of all ages. Even in countries with significant improvements in maternity services, adolescent mothers still struggle to obtain the necessary healthcare coverage. The main causes of this could be restricted access to healthcare facilities, low income, limited awareness, and living in remote areas. Another aspect is that teenage girls are less inclined to attend prenatal and postnatal care checkups [13,22,51]. Nearly a quarter of expectant adolescents do not meet the minimum requirement of at least four antenatal visits [5]. Insufficient preparation and limited access to essential information, including infant nutrition, parental care, and pediatric disease prevention, aggravate psychological stress among adolescent parents [29]. Another reason for limited health service availability is lacking insurance. In some countries, such as Rwanda, “Community-Based Health Insurance” for individuals younger than 16 needs to be purchased by their caregivers, as adolescents do not have a national identity card. In the case of adolescent pregnancy, many young girls are not married and get rejected by their families. This results in an absence of insurance and inadequate health coverage [51]. Limited access to healthcare is also reflected in the finding that 57,1% of the sampled individuals in a study conducted in Sub-Saharan African countries identified being HIV-positive as a significant concern [52].

In the end, the systematic review analyzed 45 studies on how abortion affects adolescents, and it was found that the approach of healthcare service providers significantly influenced adolescents’ well-being, perceptions of abortion, and future outlooks. Furthermore, some studies found that the sociocultural stigma and lack of information or resources led some adolescents to resort to harmful abortion practices due to uncertainty about how to handle an unplanned pregnancy. An extremely high prevalence of unsafe abortions is observed in countries where abortion is illegal and the healthcare resources are insufficient [51,53].

#### 3.3.3. The Need for Support in Areas of Stigma, Legal Rights, Education, and Finances

Unmarried and pregnant teenagers are prone to experiencing stigmatizing attitudes and discrimination from healthcare workers. Disrespect, prejudice, disdain, rejection, the feeling of victimization, and apathy may discourage adolescents from seeking professional help. This leads to young mothers experiencing insufficient pregnancy- and childbirth-related information and receiving inadequate support from the healthcare provider [5,48,51,54]. The stigma remains prevalent within the sociocultural context. Pregnant adolescents are labelled as immoral role models who must be disgraced, and the stigmatizing school environment forces pregnant girls to drop out. In addition, families, partners, and communities cease psycho-emotional and financial support for young mothers, which leads to social isolation [28,48]. Some young mothers do not always distinguish whether the stigma is aimed at being sexually active or at their decision to continue or terminate the pregnancy [55]. Despite all the challenges that come with being pregnant at a young age, harsh prevention campaigns can sometimes backfire, causing more harm than benefit. This is why advocacy groups and teenage leaders need to focus on promoting positive portrayals of adolescent sexual health and parenting. Highlighting these constructive perspectives would drive social change in a way that empowers rather than stigmatizes young people [49].

Child marriage is another widespread issue, affecting human rights. It is associated with physical and mental health deterioration, impaired self-realization, and restricted independence [44]. Another key determinant of younger childbearing age is cultural traditions, including early wedded life, widow inheritance, sororate marriage, and preference to have a son. All these elements can interact in complex ways, contributing to the likelihood of unintended pregnancies among teenagers [42,44,45]. Furthermore, in some cultures, married adolescents live in traditional extended family households where they have restricted autonomy in decision-making [13,28,35]. Adolescent pregnancy can be associated with poor socioeconomic conditions [28,42,45]. Findings suggest that girls from rural and lower-income backgrounds have a three times higher probability of marrying as a child and becoming an early parent compared to wealthier girls from urban areas. Similarly, girls with only primary education are nearly eight times more likely to experience pregnancy in adolescence compared to those who acquired higher education. Additionally, adolescents experience limited education opportunities in the case of pregnancy. In several studies, one of the main concerns was adolescent mothers’ unemployment, which reflects the financial dependence and need for supply [6,28,44,52].

#### 3.3.4. The Need for Support from Family and Community

Firstly, adolescent girls expressed the significance of maintaining a connection with the father of their baby, especially with the support of family members. Some adolescents who lived with the child’s father considered him as their primary source of support. The lack of a husband was associated with higher rates of anxiety and depression among pregnant girls. Evidence suggests that inadequate communication between parents and their children, particularly regarding SRHR, is associated with a higher risk of unintended adolescent pregnancies. Many pregnant girls struggle to cope with the overwhelming responsibility of becoming a mother. The lack of maternity knowledge and immaturity highlights the need for guidance and support. In addition to family, they also relied on teachers, social workers, school nurses, and other pregnant or parenting peers for help [6,27,39,52].

The most common need among peripartum adolescents in Sub-Saharan Africa is emotional support from the community, as reported by 85.7% of individuals [52]. On the other hand, support from healthcare workers is as important. Unbiased communication and compassion from hospital staff were crucial for expectant mothers to feel comfortable throughout their pregnancy and labor. They also wanted to feel emotionally connected with nurses and doctors during labor [43].

Additionally, unplanned pregnancy at a young age emphasizes adolescents’ need to develop coping strategies involving internal resources and social support. Pregnant adolescents require unique coping approaches to manage their sense of helplessness. It is important to foster youths’ problem-solving abilities and involve family support [27,29,32]. Participants shared a desire to connect with others who had experienced an unintended pregnancy at a young age. They wanted to ask questions to someone who had similar experience and would not judge them. Almost all participants believed such connections would lessen their isolation and stigma, offering the answers and support they needed during that time [55].

### 3.4. Support Systems for Adolescent Mothers

The review of the included references highlighted some interventions and support strategies for pregnant adolescents.

#### 3.4.1. Contraception and Reproductive Health Education

The high number of teenage pregnancies reflects issues in sexual education and contraceptive behavior. In the United States of America, ~70% of youth engage in sexual intercourse before the age of 19 [4]. Considering the widespread prevalence, A. Lewin et al. investigated the impact of the Generation program on this issue in 2016. It is one of Washington’s Patient-Centered Medical Home interventions, providing care and reproductive health services for adolescent parents and their offspring. The principal components of this program are family-focused primary care, holistic social work support, and mental health evaluation and management. The analysis of 12-month follow-up data showed that participation in the Generations initiative is associated with the improved use of contraception. Adolescents enrolled in this program were almost three times more likely to use contraception and did so in a more consistent manner than the control group. The American Academy of Pediatrics additionally recommends expanding long-acting reversible contraception education and increasing its provisions, as it is a first-choice pregnancy prevention method for adolescents. The evidence suggests that prescribing proper birth control to adolescents in the early postpartum period effectively prevents repeated pregnancy [17,46].

Another approach is the “Champions of Change” program, implemented in 2022 by Epworth and Overspill Clinics in the district of Zimbabwe. The initiative focuses on providing reproductive and maternal health education for adolescent mothers and pregnant peers. In 6 months of involvement in this project, use of contraception, knowledge about sexual and reproductive rights, and access to sexual gender-based violence centers significantly improved. According to the research, healthy parenting strategies for pregnant adolescent caregivers improved family relationships and resulted in better sexual and reproductive health outcomes. Additionally, a respectful patient–healthcare worker relationship contributed to better contraceptive behavior [48].

Effective and unbiased sex education involving both genders significantly reduces the likelihood of pregnancy in adolescent years.

#### 3.4.2. High-Quality and Accessible Healthcare Possibilities

Acknowledging the high prevalence of unintended pregnancies, many professional organizations, like the United States Preventive Services Task Force or the Centers for Disease Control and Prevention, advocate for preconception counselling for all women of reproductive age starting from their teenage years. The importance of this is in evaluating potential risk factors and taking appropriate actions to prevent negative fetal and maternal outcomes. Some of the key aspects of it are chronic illness management, alcohol, tobacco, and other substance abuse reduction, immunization, and supplementation. The US Preventive Service Task Force and other societies recommend that all healthcare specialists advise young girls to take supplements containing folic acid after menarche and promote reproductive health education [50].

Another example of a medical center organizing support for pregnant adolescents is the Teen and Tot Program (TTP) at Boston Medical Center’s Adolescent Center. The program focuses on adolescent pregnancy and parenting, providing care for both teen parents and their children in one visit and setting. This model serves as an early example of the patient-centered medical home, where increased provider contact with teen parents helps simplify access to contraceptives and places their use within a broader context of family health [56].

Unfavorable interactions between patients and medical workers affect the healthcare outcome. Negative care providers’ attitudes and ineffective communication can worsen patients’ mental health satisfaction with care, restrict access to information, increase the sense of fear and mistrust [5,48]. That is why enhancing healthcare workers’ ability to accept, support, and respect patients is crucial to fostering non-discriminatory interactions. The South African National Department of Health evolved the South African Adolescent Youth-Friendly Services approach, which implements education programs for healthcare providers. Adolescent-friendly services still require improved training, development of national guidelines, implementation of behavioral change interventions, and a clarification of values [5].

Some studies highlight the importance of regular psychological assessment of pregnant adolescents, detecting at-risk patients, and providing necessary assistance. The mental health burden can be mitigated through accessible and free consultations with mental health specialists [26,35,51,52]. Mental health education is usually neglected but essential to reduce the psychological burden of pregnant adolescents. A study conducted in primary healthcare facilities in Sleman, Yogyakarta found that the *Mother and Child Health Handbook* was an accessible and effective tool for young mothers. The illustrations were identified as a key feature that facilitated understanding among pregnant Indonesian adolescents. This finding reinforces the idea that integrating visual or media-based tools can enhance adolescent mothers’ understanding of mental and physical health-related information [27].

#### 3.4.3. Regular Assistance from the Community, Professionals, and Educational Programs

The Young Parents Project, established in Florida state, provides intensive support for high-need young mothers through multidisciplinary services. Focusing on the mother-baby relationship ensures safety, personalized care, and respect for cultural values. Weekly home visits of social workers foster family engagement, assessment, and access to community resources. Additionally, a social worker helps teenagers navigate the court process, ensuring they understand proceedings and have a voice in court [49]. Integrated effective coping mechanisms and parenting programs show young girls how to survive and take proper care of their children. New habits and friendships help to replace the feeling of social stigma and contribute to even greater resilience, self-confidence, and lower psychological stress. Moreover, the risk of negative adolescent pregnancy outcomes can be minimized by implementing in-home mental health educational interventions involving family members, midwives, nurses, and other professionals. Psychological readiness and an optimistic outlook are fundamental for adolescents’ well-being and ability to encounter difficulties in assuming motherhood [6,17,27,29,32,51]. Another significant implementation would be adolescent mentorship initiatives that encourage teenagers to pursue academic qualifications and employment. Moreover, it is important to incorporate interventions to reduce substance abuse [17,28].

It is also essential to enhance young women’s self-efficacy, strengthen their resistance to sexual pressure, and develop personal intentions to omit pregnancies during adolescence [17]. Non-profit organizations could offer small financial resources to launch businesses, cover school fees for adolescents who wish to continue their education, and organize skill workshops for those who aspire for an improved future for themselves and their offspring [29]. Some healthcare centers facilitate group meetings for adolescent mothers, creating a secure environment for psycho-emotional support [51]. In Turkey, obstetrics and gynecology outpatient clinics apply a psychosocial support-based psychoeducation program for pregnant adolescents, consisting of eight interactive sessions over 4 weeks. The program focuses on coping strategies, emotional regulation, and social support networks, with culturally relevant interventions emphasizing family and community involvement. Results showed a significant decrease in anxiety and depression levels, along with a significant increase in perceived social support levels in the experimental group compared to the control group after the psychoeducation intervention [57].

**Figure 2:**
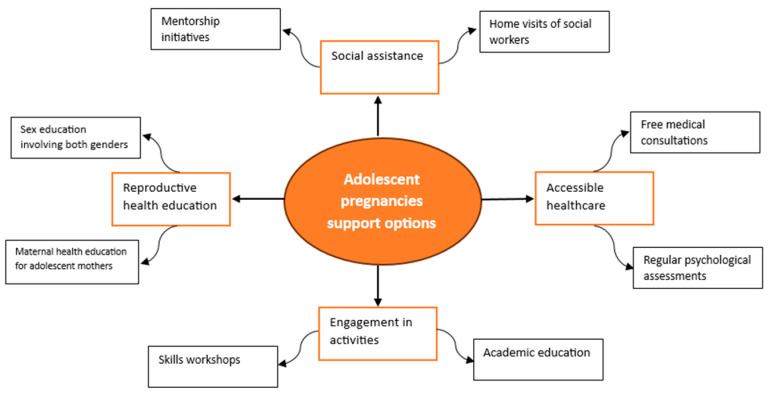
Mind map of possible support options for adolescent parents during the perinatal period.

In summary, Figure 2 presents various approaches to ensuring comprehensive support for adolescent parents during the perinatal period, which is crucial for their and their children’s well-being.

### 3.5. Challenges and Gaps in Current Support Systems

Significant gaps exist, including in family, school, community, social, and legal systems. Formal support groups for teenage mothers or pregnant teens are scarce at the national level, but many women express that connecting with other young pregnant women—whether through online communities or personal networks—has a positive and empowering impact on their experience with unintended pregnancy. However, psychologist-led formal groups could provide reliable information and foster healthy connections among teenage mothers [58]. Healthcare providers face challenges balancing parental involvement and adolescent confidentiality in sexual and reproductive health services. The use of electronic health records complicates this issue, as parents push for continued access to their child’s records, potentially compromising confidentiality. Providers navigate this tension in different ways, such as selectively sharing visit summaries with adolescents [58]. Healthcare providers still struggle with their attitudes toward adolescent pregnant girls. While they effectively engage with young mothers in managing depression, their views on adolescent pregnancy remain largely unchanged [59]. Another gap in current support systems is that parents of pregnant adolescents lack external support when it comes to communicating with their children about sex and sexuality. It is assumed that discussions about sex and sexuality are seen as conflicting with the concept of “respect” in the parent–child relationship. Inadequate recognition of non-consensual sexual experiences is another gap to consider. The fact that many girls became pregnant through rape in the context of “boyfriend–girlfriend” relationships or at the hands of relatives, friends, or strangers reflects a need for more education, protection, and awareness about consent and healthy relationships [47]. It is essential to investigate legal issues concerning sexual abuse, educate young girls about their rights, and promote seeking help or support. The government needs to ensure vulnerable populations that sexual assault perpetrators will be convicted and all of the victims’ rights will be protected [32]. Healthcare workers at Mitundu Rural Hospital in Malawi highlight the lack of culturally appropriate screening tools to assess common mental health disorders during routine evaluations. Many adolescent mothers with mental health concerns remain unrecognized, especially if they show no visible signs of sadness. Additionally, participants noted that the hospital had only one mental health nurse in the labor ward and just two nurses to care for fifty mothers daily, with only two nurses per shift [60].

## 4. Strengths and Limitations

This study has several strengths. We systematically compiled and organized key findings into tables categorizing the main mental health challenges and needs of adolescent mothers. Additionally, beyond identifying the problem, we provided examples of support initiatives and interventions. By incorporating global data from various countries, we ensured a comprehensive perspective on the prevalence of the issue. Furthermore, we included Lithuanian data, which allowed for us to highlight the limited attention given to adolescent pregnancy in Lithuania and the lack of specialized support services for pregnant minors. However, some limitations should be acknowledged. We did not specifically address the topic of abortion, as it was not included as a keyword in our search strategy. Additionally, we did not review long-term mental health consequences or interventions beyond the perinatal period, which could be valuable areas for future research. Despite our efforts to provide a global perspective, certain regions may be underrepresented due to a lack of data availability.

## 5. Future Directions

Difficulties related to adolescent pregnancy should be addressed not only through psychological health support, but also within a nurturing, cooperative, and tolerant environment. In addition, the approach should involve legal institutions, reproductive health consultations, and community-based initiatives. Healthcare workers have to apply evidence-based practices to support adolescent motherhood, reduce psychological pressure, improve confidence, educate on infant care, and address gender inequality and sociocultural stigma. The community must shift its negative perception of adolescent pregnancy and be more supportive and empathetic toward girls facing the challenge of motherhood. It is important to raise awareness and provide more effective sources of information or coping strategies in case of possible psychological well-being deterioration during the perinatal period. Although the reviewed literature illustrates several successful options of supporting initiatives, a lack of compelling examples remains. More effective support strategies are crucial for bridging the gaps in the current system.

One more identified shortcoming is that most of the literature scopes African, Asian, and American data. Given the severity of the issue in the regions mentioned before, this finding is reasonable. However, a more precise analysis of adolescent pregnancy and perinatal mental health in European countries is needed. Lithuania is not the exception. As we found only a limited number of Lithuanian references on this issue, it underscored the need for a deeper exploration of adolescent pregnancy, perinatal mental health, associated needs, and comprehensive support options in our country. However, we did not emphasize the information regarding Lithuania, as our primary focus was on the global scale of the problem rather than its extent within our country.

Another serious hardship is abortion laws. Currently, several governmental and public debates worldwide are focused on the topic of abortion. Negotiations encompass various ethical, legal, cultural, religious, and medical aspects, with no clear consensus reached. We suggest prioritizing a comprehensive approach for unintended adolescent pregnancies because these are associated with higher risks, such as unsafe abortions, as discussed above.

## 6. Conclusions

This literature review emphasizes the mental health issues, needs, and complex support options of pregnant adolescents while also highlighting the challenges and gaps in the current system. Pregnancy in adolescence carries a higher risk compared to pregnancy in adult years. Perinatal mental health management in young teenagers requires a multidisciplinary approach to address their specific challenges. This can contribute to shaping teenage parenthood with dignity, focusing on family strengths, and offering nonjudgmental support. An unexpected adolescent pregnancy can be reframed as a meaningful conversion to maturity, where they strive to parent despite facing mental health difficulties, stigma, and having limited resources. In conclusion, adolescent pregnancy is a sensitive psychosocial issue with relatively few scientifically proven support methods. Currently, assistance is provided only through isolated initiatives, with no comprehensive national support system in place. When developing support structures, it is essential to consider the cultural characteristics of the country to ensure their effectiveness and acceptance within society.

## Figures and Tables

**Table 1:** Summary characteristics of adolescent pregnancy prevalence studies.

Authors	Study Design	Analyzed Period	Countries	Race/Nationality	Age Group (Years) and/or Mean Age ± SD	Rate (Per 1000 People)
Martin et al., 2019 [15]	Population-Based Registry Study	2016	United States	White, Black, American Indian or Alaska Native, Asian, Native Hawaiian or Other Pacific Islander, Hispanic	15–19	20.3
Ojukwu et al., 2024 [17]	A scoping review	2010–2023	North America	African, Caribbean, and Black North American	10–19	1.9–93.5
Eminov et al., 2025 [19]	A retrospective study	2020–2023	Turkey	Turkish	≤19 18.23 ± 0.880	101.2
Tretjakova et al., 2018 [22]	Population-Based Registry Study	2015	European union	Austrian, Belgian, British, Bulgarian, Croatian, Cypriot, Czech, Danish, Estonian, Finnish, French, German, Greek, Hungarian, Irish, Italian, Latvian, Lithuanian, Luxembourgish, Maltese, Dutch, Polish, Portuguese, Romanian, Slovak, Slovenian, Spanish, Swedish	15–19	10.8
Varmaghani et al., 2024 [23]	A systematic review and meta-analysis	1995–2020	Iran, Saudi Arabia, Iraq, Oman, Lebanon, Jordan, Pakistan	Iranian, Saudi Arabian, Iraqi, Omani, Lebanese, Jordanian, Pakistani	10–19	90
Mebrahtu et al., 2024 [10]	An observational cohort study	2017–2019	KwaZulu-Natal, South Africa	African	13–18	10–130
Kassa et al., 2018 [25]	A systematic review and Meta-analysis	2002–2018	24 African countries from East, West, Southern, Central and Northern sub-regions	African	10–19	188

**Table 2:** Summary of studies that investigated mental health issues.

Author	Subject	Population Source	Study Design	Reference
Adolescent Perinatal Mental Health Issues				
Corcoran, 2016	Depression	Adolescent women during pregnancy and the postpartum period	Literature review	[34]
Kassa et al., 2023		Adolescents aged 10–19 years, six weeks after childbirth from northwest Ethiopia	Cross-sectional	[25]
Sakakibara et al., 2024		Adolescents (15–19 years old) who were pregnant (>3 months) or had recently given birth (<3 months) at Tororo District Hospital in Uganda	Cross-sectional	[28]
Mohammad et al., 2021		Women aged less than 20 years, six to eight weeks postpartum, and who could speak and read Arabic	Cross-sectional	[35]
Rahim et al., 2024		Adolescent mothers beyond the postpartum period from the United States, France, Australia, Bangladesh, the United Kingdom, South Africa, and Thailand	A systematic review and meta-analysis	[36]
Gebrekristos et al., 2025		Adolescent mothers ≤1 year postpartum (aged 14–19 years old) in an informal settlement in Nairobi, Kenya	Cross-sectional	[11]
Patiño et al., 2024		Adolescent mothers aged 14 to 19 from Mexico City	Cross-sectional	[37]
Oladeji et al., 2022		Pregnant adolescents presenting to primary maternal and child healthcare centers in Ibadan, Nigeria	Randomized controlled trial	[38]
Otika et al., 2024		Pregnant and non-pregnant adolescents between 15 and 19 years old living in the refugee settlements of northern Uganda	Cross-sectional descriptive observational study	[32]
Asante et al., 2024		Adolescent pregnant women visiting antenatal clinics in Ghana	Cross-sectional study	[31]
Hymas et al., 2019		Adolescent mothers <20 years of age with onset of illness within 12 months of childbirth	A systematic review	[9]
Recto et al., 2018		Teenagers self-identified as Mexican American either pregnant or postpartum (up to 1 year)	Qualitative description study	[39]
Otika et al., 2024	Suicide	Pregnant and non-pregnant adolescents between 15 to 19 years old living in the refugee settlements of northern Uganda	Cross-sectional descriptive observational study	[32]
Rahim et al., 2024		Adolescent mothers beyond the postpartum period from the United States, France, Australia, Bangladesh, the United Kingdom, South Africa, and Thailand	A systematic review and meta-analysis	[36]
Gelaye et al., 2016		Adolescent girls during pregnancy from the United States, Pakistan, Brazil, Peru, Italy, Finland, and Bangladesh.	Systematic review	[40]
Gebrekristos et al., 2025	Low self-esteem	Adolescent mothers ≤1 year postpartum (aged 14–19 years old) in an informal settlement in Nairobi, Kenya	Cross-sectional	[11]
Tenaw et al., 2024		Teenage mothers from the United States, Indonesia, Uganda, Ghana, the Philippines, Australia, and South Africa	A qualitative meta-synthesis	[6]
Sakakibara et al., 2024		Adolescents (15–19 years old) who were pregnant (>3 months) or had recently given birth (<3 months) at Tororo District Hospital in Uganda	Cross-sectional	[28]
Recto et al., 2018	Using substances	Teenagers self-identified as Mexican American either pregnant or postpartum (up to 1 year)	Qualitative description study	[39]
Ojukwu et al., 2024		Adolescents aged 10–19 from the United States	A scoping review	[17]
Peter et al., 2016	Anxiety	Pregnant women aged 10 to 19 years who received prenatal care in southern Brazil	Cross-sectional study	[41]
Osok et al., 2021		Pregnant adolescents (ages 15–19) visiting a health facility’s antenatal services in Nairobi	Cross-sectional survey study	[42]
Kazal et al., 2021		Adolescent mothers (age 15–20 years at time of recruitment) and within 1 year of delivery in Rhode Island	Mixed quantitative and qualitative study	[43]

**Table 3:** Summary of categorized complex needs.

Categories of Needs	Pregnant Adolescents’ Needs	Number of Studies	References
Sexual and reproductive health and rights	Contraceptive education; Protection from sexual and gender-based violence or abuse; Healthy sexual relationship education; Encouragement to disclose harmful experiences; Development of available sexual and reproductive health services.	7	[28,42,44,45,46,47,48]
High-quality and accessible healthcare possibilities	Available methods of contraception; Discussing reproductive health with a primary care physician; Preconception preventive care strategies for adolescents; Advanced mental health services; Accessible healthcare in rural and low-income areas; Available healthcare insurance for pregnant adolescents; Comprehensive perinatal healthcare guidance; Accessing safe and legal abortion.	11	[5,13,22,27,29,44,49,50,51,52,53]
Support in areas of stigma	Reduced stigma in healthcare settings; Reduced stigma in society; Advocacy for positive adolescent sexual health and parenting representations.	7	[5,28,48,49,51,54,55]
Support in areas of children’s rights	Prevention of child marriages; Restriction of cultural traditions violating children’s rights; Adolescents’ autonomy protection.	6	[13,28,35,42,44,45]
Education and economic support	Financial support; Available education possibilities during or after pregnancy; Employment opportunities.	6	[6,28,42,44,45,52]
Support from family and community	Connection with the baby’s father; Reliable communication between adolescents and their parents; Guidance on maternity from family, teachers, social workers and school nurses; Emotional and compassionate connection with healthcare workers and community members; Development of coping strategies for young girls; Solidarity from pregnant or parenting peers.	8	[6,27,29,32,39,43,52,55]
